# *Origanum vulgare* Essential Oil vs. a Commercial Mixture of Essential Oils: In Vitro Effectiveness on *Salmonella* spp. from Poultry and Swine Intensive Livestock

**DOI:** 10.3390/antibiotics9110763

**Published:** 2020-10-31

**Authors:** Maura Di Vito, Margherita Cacaci, Lorenzo Barbanti, Cecilia Martini, Maurizio Sanguinetti, Stefania Benvenuti, Giovanni Tosi, Laura Fiorentini, Maurizio Scozzoli, Francesca Bugli, Paola Mattarelli

**Affiliations:** 1 Dipartimento di Scienze e Tecnologie Agro-Alimentari, Università di Bologna, Viale G. Fanin 42, 40127 Bologna, Italy; wdivit@gmail.com (M.D.V.); lorenzo.barbanti@unibo.it (L.B.); paola.mattarelli@unibo.it (P.M.); 2 Dipartimento di Scienze Biotecnologiche di Base, Cliniche Intensivologiche e Perioperatorie, Università Cattolica del Sacro Cuore, Largo A. Gemelli 8, 00168 Rome, Italy; margherita.c86@gmail.com (M.C.); ceciliamartini84@gmail.com (C.M.); francesca.bugli@unicatt.it (F.B.); 3 Dipartimento di Scienze di Laboratorio e Infettivologiche, Fondazione Policlinico Universitario A. Gemelli IRCCS, Largo A. Gemelli 8, 00168 Rome, Italy; 4 Dipartimento di Scienze della Vita, Università di Modena e Reggio Emilia, Via G. Campi 103, 41125 Modena, Italy; stefania.benvenuti@unimore.it; 5 Istituto Zooprofilattico Sperimentale della Lombardia e dell’Emilia-Romagna “Bruno Ubertini”, Sede Territoriale di Forlì, Via Don E. Servadei 3E/3F, 47122 Forlì, Italy; giovanni.tosi@izsler.it (G.T.); laura.fiorentini@izsler.it (L.F.); 6 Società Italiana per la Ricerca sugli Oli Essenziali (SIROE), Viale Regina Elena 299, 00161 Rome, Italy; mscozzoli@gmail.com

**Keywords:** *Salmonella*, *Origanum vulgare*, ciprofloxacin, poultry farms, pig farms

## Abstract

*Salmonella* spp. represent a public health concern for humans and animals due to the increase of antibiotic resistances. In this scenario, the use of essential oils (EOs) could be a valid tool against *Salmonella* contamination of meat. This work compares the in vitro effectiveness of an Italian mixture of feed additives based on EOs (GR-OLI) with EO of *Origanum vulgare* L., recently admitted by European Food Safety Authority (EFSA) for animal use. Twenty-nine *Salmonella* serotypes isolated from poultry and pig farms were used to assess GR-OLI and *O. vulgare* EO antimicrobial propeties. *O. vulgare* EO was active on the disaggregation of mature biofilm, while GR-OLI was capable of inhibiting biofilm formation and disaggregating preformed biofilm. Furthermore, GR-OLI inhibited bacterial adhesion to Caco-2 cells in a dose-dependent manner. Both products showed inhibition of bacterial growth at all time points tested. Finally, the synergistic action of GR-OLI with commonly used antibiotics against resistant strains was investigated. In conclusion, the mixture could be used both to reduce the meat contamination of *Salmonella* spp. before slaughter, and in synergy with low doses of ciprofloxacin against resistant strains. Although EOs as feed additives are already used in animal husbandry, no scientific study has ever highlighted their real antimicrobial potential.

## 1. Introduction

The serotypes of *Salmonella* spp, a pathogenic genus of Enterobacteriaceae, are responsible for animal infections from a sub-clinical to severe level, as well as typhoid fever and severe diarrhea in humans [1]. These Gram-negative bacteria are one of the major causes of concern for the veterinary industry. In particular, chickens and pigs are known as *Salmonella*’s major vehicles. Great efforts are aimed at controlling the *Salmonella* spp. colonization in pig and chicken reservoirs since these animals, if infected, generally host the bacterium asymptomatically in the tonsils, intestine, and lymphoid tissue associated with the intestine [2]. According to the community summary report on trends and sources of zoonoses and zoonotic agents and foodborne outbreaks in the European Union [3], the three most commonly reported zoonoses in Europe are the foodborne enteric diseases campylobacteriosis, salmonellosis, and yersiniosis. Recent studies have identified *Salmonella* spp. multi-resistance to the most common antibiotics used in livestock [4,5]. For this reason, the European Food Safety Authority (EFSA) considers *Salmonella* serotypes isolated from livestock as a danger to public health [6], and the European Union banned the use of antibiotics in animal food production as growth promoters [7]. Subsequently, control measures are aimed at reducing the prevalence of *Salmonella* spp. in livestock, especially in chickens and pigs, and the search for valid alternatives to the use of antibiotics has been stimulated. Probiotics, prebiotics, acidifiers, plant extracts, and nutraceuticals are new alternatives to antibiotics that are widely investigated by researchers [8,9]. Among them, essential oils (EOs) have a prominent place [9].

According to the International Organization for Standardization, essential oils are volatile products obtained from “natural raw material of plant origin by steam distillation, by mechanical processes from the epicarp of citrus fruits, or by dry distillation, after separation of the aqueous phase—if any—by physical processes” [10].

To date, many studies have evaluated the in vitro and in vivo efficacy of EOs on livestock microbial strains, including Salmonella spp. Several studies have analyzed the effects of EOs or their preparations on live animals from pig and poultry farms [11,12,13], on strains isolated from farms, on reference strains [14], and even on meat after slaughtering [15,16,17,18], in order to reduce the infection of Salmonella spp. or increase the shelf-life of the products intended for human consumption. However, not all EOs have been shown to be suitable for in vivo use, as the minimum inhibitory concentration (MIC) of most EOs is much higher than the acceptable dose levels in animal industry in terms of cost-effectiveness and feed palatability [9].

In November 2019, the EFSA published a report expressing the Agency’s Endorsement of the safety and efficacy of *Origanum vulgare* L. EO use in feed of all animal species [19]. For each animal species, this report indicates the allowed dosages of the EO expressed in mg/kg live weight. The doses of 22 mg/kg for fattening chickens, 33 mg/kg for laying hens, 30 mg/kg for fattening turkeys, 40 mg/kg for piglets, and 48 mg/kg for fattening pigs are established as safe for both humans and animals, and they are not expected to pose a risk for the environment. According to European Commission Regulation No 1334/20083, *O. vulgare* EO can be used as a flavouring additive in all animal feed, without additional evaluation and approval [4]. The antimicrobial action of *O. vulgare* EO on both bacteria and fungi has been documented in several studies. Generally, the antimicrobial action of this EO is more effective in fungi than it is in bacteria. More specifically, it is more effective against Gram-positive bacteria than Gram-negative bacteria [20]. The EO of *O. vulgare* L. acts on fungal cells by thinning the morphology of the hyphae, and inducing an oxidative stress until cell lysis [21,22]. In bacteria, the main target of the EO active chemicals is the cellular phospholipid bilayer [23]. In particular, the two major chemicals of the EO (thymol and carvacrol) disrupt the outer membrane, alter the proton gradient, and inhibit the production of ATP of Gram-negative cells (including *Salmonella* spp cells) [24,25]. Carvacrol is the principal active compound of the *O. vulgare* phytocomplex, and it belongs to phenols that could exert toxic effects.

The aim of this study was to compare the in vitro antimicrobial effectiveness of the *O. vulgare* EO vs. a mixture of feed additives, namely GR-OLI, characterised by a lower concentration of carvacrol and already approved for use in animal feed, on 29 strains of *Salmonella* spp. isolated from poultry and pig farms.

## 2. Results

### 2.1. Compositional Analysis

The constituents identified from *O. vulgare* EO and GR-OLI, their linear retention indices (LRIs), and their percent composition are reported in Table 1. Specifically, the EO of *O. vulgare* is composed of 66.98% carvacrol, while the GR-OLI mixture is characterized by four major compounds with concentrations greater than 10% (limonene 15.32%, carvacrol 12.50%, 1–8 cineol 11.95%, and p-cymene 10.62%).

### 2.2. Antibiograms

All *Salmonella* spp. strains were tested for their sensitivity against antibiotics commonly used in human medicine. As indicated in Table 2, all *S.* Typhimurium strains, with the exception of the monophasic variant, were sensitive to most antibiotics except amoxicillin/clavulanic acid and ciprofloxacin, to which about 31% (S24, S31, S32 S34) and 54% (S3, S7, S12, S17, S18, S21 S32) of strains were resistant, respectively. Only the S17 and S32 strains were also resistant to gentamicin, while S24 and S31 were also resistant to trimethoprim/sulfamethoxazole. All monophasic *S.* Typhimurium strains were resistant to amoxicillin/clavulanic acid; S19 and S28 strains were resistant to ciprofloxacin, and only S29 to trimethoprim/sulfamethoxazole. Whereas, S19 and S29 strains were susceptible by increased exposure sensitivity to piperacillin/tazobactam and ceftazidime, respectively. The S13 strain was sensitive to all antibiotics tested. *S.* Infantis strains showed various multi-resistances. Specifically, no strain was resistant to piperacillin/tazobactam, ertapenem, imipenem, meropenem, amikacin, and gentamicin, while all strains were resistant to ciprofloxacin. In total, 75% of the tested strains (i.e., all excluding S10, S26, and S42) were resistant to trimethoprim/sulfamethoxazole, 67% (all except S4, S10, S40, and S42) to amoxicillin/clavulanic acid, 58% (all minus S4, S25, S38, S39, and S42) to cefotaxime, and 50% (all apart S4, S10, S25, S38, S39 and S42) to cefepime. Increased exposure sensitivities were shown by the S10 strain vs. cefepime, and by 58% of the strains vs. ceftazidime (i.e, all strains except S4, S25, S38, S39, and S42).

### 2.3. Broth Microdilution Susceptibility Testing

The broth microdilution susceptibility test was performed to study the in vitro sensitivity of *Salmonella* spp. vs. *O. vulgare* EO and GR-OLI. As shown in Table 1, the two natural products have the same activity against both *S.* Typhimurium and *S.* Infantis. In particular, the *O. vulgare* EO and the GR-OLI have a respective minimal inhibitory concentration of the 90% of strains (MIC90) equal to 2% *v/v* (20 μL/mL) and 16% *v/v* (equal to 40 μL of EOs content /mL), for both *S.* Typhimurium and *S.* Infantis.

### 2.4. Biofilm Formation Assay

**Figure 1:**
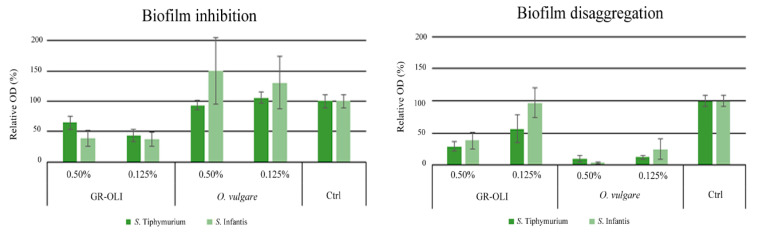
Control-related ratios of the OD values measured for *S.* Typhimurium and *S.* Infantis biofilm inhibition and biofilm disaggregation by GR-OLI and *O. vulgare* EO. Vertical bars indicate ± standard errors.

The ability of low concentrations of *O. vulgare* EO and GR-OLI to inhibit bacterial biofilm formation or disaggregate a preformed biofilm was evaluated. As shown in Figure 1, the two concentrations tested of GR-OLI were capable of inhibiting biofilm formation or disaggregating any preformed biofilm for *S.* Typhimurium. Both concentrations showed activity in the inhibition of the biofilm formation of *S.* Infantis, but only the higher concentration was active in disaggregating the preformed biofilm. *O. vulgare* EO was active only in the disaggregation of mature biofilm of both serotypes, but no inhibiting activity was exerted on biofilm formation.

### 2.5. Cell Adhesion Assay

**Figure 2:**
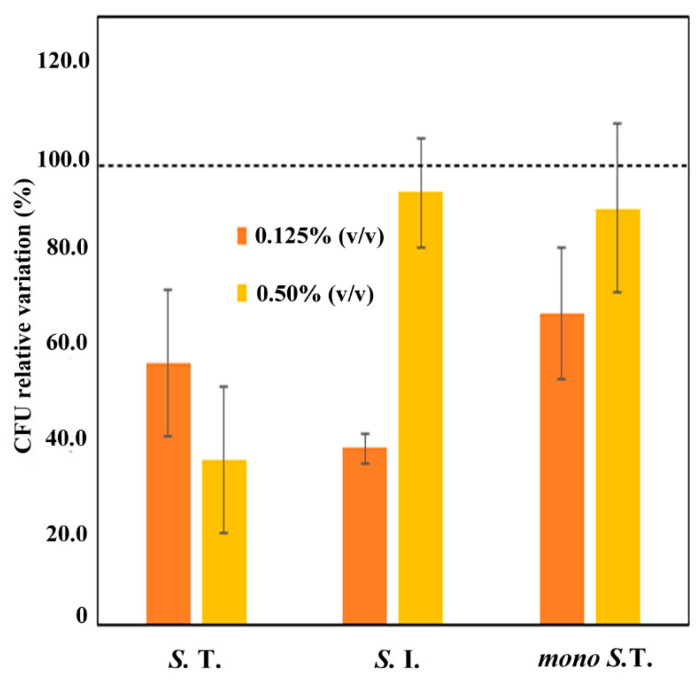
Control-related ratios of the CFU count recovered from GR-OLI-treated bacteria strains (S.T. = *S.* Typhimurium, S. I. = *S.* Infantis, mono S. T. = monophasic *S.* Typhimurium) after the adhesion to the Caco-2 monolayer. Vertical bars indicate ± standard errors.

Since GR-OLI was effective not only in the disaggregation of the preformed biofilm but also on the inhibition of biofilm formation, we decided to investigate the interference of this natural mixture on bacterial adhesion to target cells. An adhesion assay was performed using eight *Salmonella* spp. strains. In particular, two *Salmonella* spp. strains sensitive to almost all antibiotics (one sensible *S.* Typhimurium and one *S.* Infantis resistant only to ciprofloxacin), and six multi-resistant strains (two *S.* Typhimurium strains, two monophasic *S.* Typhimurium, and two *S.* Infantis strains) were tested. Figure 2 shows the control-related ratios of the colony-forming unit (CFU) count obtained after the adhesion of the bacteria strains, pre-treated with GR-OLI and untreated, to the Caco-2 monolayer. The ratios refer to bacteria that are still able to adhere to Caco-2 cells after treatment with GR-OLI. Data show that *S.* Typhimurium strains were inhibited in a proportion to the GR-OLI concentration. Particularly, the inhibition was significant at the lower concentration for both monophasic and non-monophasic *S.* Typhimurium (bars of standard error not attaining the 100% level of the control), while the higher concentration was effective only on non-monophasic *S.* Typhimurium. In reverse, the only significant inhibition of *S.* Infantis adhesion was obtained at the minimal concentration tested.

### 2.6. Growth Curves

The same strains selected for the cell adhesion assay were treated with GR-OLI and *O. vulgare* EO to evaluate the impact of the two compounds on the bacterial growth curve. Growth curves showed that both products, when tested at the first sub-MIC concentrations, were able to inhibit the growth of most of the tested *Salmonella* strains. Furthermore, the inhibition determined by *O. vulgare* EO on *S.* Typhimurium strains was significantly stronger than that of the GR-OLI at 15 and 20 h (Table 3). Whereas, if compared to the control (Ctrl), both products showed an equal and significant inhibition of *S.* Infantis growth at all the time points tested.

### 2.7. Checkerboard Titration Method

The four strains (S26, S35, S36, S37) equally resistant to amoxicillin/clavulanic acid, cefotaxime, and ciprofloxacin were chosen to test the capability of GR-OLI to synergically reactivate the sensitivity to one or more of these drugs. Table 4 shows the values of the minimal bactericidal concentration (MBC) of each single compound, those of the GR-OLI/antibiotics interaction, and the relative values of the fractional bactericidal concentration index (FBCI). Due to the turbidity of the wells containing both the dilutions of the GR-OLI and those of the antibiotic, it was not possible to perform the OD450 to obtain the MIC values and the relative fractional bactericidal concentration (FIC) index. Ciprofloxacin is the only antibiotic showing synergy with the GR-OLI for all the samples analyzed, while amoxicillin/clavulanic and cefotaxime had a variable interaction depending on the strain analyzed.

## 3. Discussion

*Salmonella* is the second human bacterial zoonosis delivered especially by chicken and pork meats, but also milk, eggs, and seafood. The WHO estimates 550 million people (including 220 million children under the age of 5 years) fall ill each year due to diarrhoeal diseases due to unsafe food [26]. In 2018, 91,857 confirmed cases of salmonellosis in humans were reported with an EU notification (EFSA, 2019), while the U.S. Center for Disease Control and Prevention (CDC) estimates that *Salmonella* bacteria cause about 1.35 million infections per year in the United States, of which only 41,930 in 2011 were laboratory confirmed [27,28]. Along with the world population increase, the consumption of meat is also increasing. Foley et al. [19] reported that since the early 1900s, the consumption of chicken in the U.S. has increased about sixfold, while pork consumption by about 20%. Whereas, the European Union data show that in 2018, Europe increased its chicken meat production by a quarter, and 70% of this production was in six member states: Poland (16.8%), the United Kingdom (12.9%), France (11.4%), Spain (10.7%), Germany (10.4%), and Italy (8.5%) [18]. An upward trend, although less steep than in the case of poultry meat, was recorded for pork meat whose consumption, in Europe, has increased by about 3.5% per person in 10 years [19]. In order to meet consumer demands, unavoidable changes in animal production were necessary. The introduction of intensive animal husbandry practices has on the one hand increased the exposure of consumers to zoonosis, and, on the other hand, has probably modified the characteristics of *Salmonella* spp. colonization in farms by selecting strains resistant to antibiotics. In animals, *Salmonella* infection can cause fever, diarrhea, prostration, and mortality. Most of the animals that survive this infection remain asymptomatic carriers, posing a threat to human health as, during slaughtering, their carcasses can contaminate others [20,21]. Within *Salmonella* serotypes, *S.* Typhimurium, *S.* Enteritidis, *S.* Heidelberg, *S.* Montevideo, and *S.* Infantis are among the major pig and poultry serotypes most frequently associated with human infections [1]. Strains of *Salmonella* spp. with antimicrobial drug resistance acquired in the animal host are now widespread in all countries [22]. Resistance to ciprofloxacin, which belongs to the group of fluoroquinolones and was, until the last decade, the treatment of choice, and to cephalosporins is increasingly being documented [22,23,24,25]. Therefore, the WHO listed resistant *Salmonella* spp. among priority pathogens for which new antibiotics were urgently needed, and several countries have established *Salmonella* surveillance and control programmes. Our data agree with the above-reported concern, because the presence of widespread resistance to ciprofloxacin is confirmed by the circumstance that 21 of the 29 analyzed strains (72.4%) were resistant to fluoroquinolone, and highlight the resistance or a reduced sensitivity to cephalosporins (cefotaxime, ceftazidime, and cefepime), especially in the *S.* Infantis serotype. Amoxicillin/clavulanic acid is another drug showing decreased efficacy especially against *S.* Infantis and monophasic *S.* Typhimurium strains. Although the combination of amoxicillin with clavulanic acid overcomes the intrinsic resistance of beta-lactamase-producing strains, and therefore makes it one of the main antimicrobial substances in swine medicine for the treatment and control of infections, the fair percentage of resistance (55.2% of strains) supports the choice of the European Medicine Agency [26] to classify this association in category C. This category includes antibiotics that are approved for use in livestock and pet animals, but which must be used with caution, only when there are few or no alternatives belonging to category D [26,27]. Natural substances represent a valid resource in the search for alternatives to current antibiotics. Thanks to their high antimicrobial potential, EOs are widely studied to counteract the development of antibiotic resistance and respond to the growing demand of consumers for antibiotic-free foods [12,28]. As noted in the introduction, the *O. vulgare* EO was found to be active against a broad spectrum of microorganisms. The antimicrobial activity is essentially mediated by the main chemicals carvacrol and thymol, which, because of their amphipathic nature, interact with the bacterial and fungal cell membrane. In particular, carvacrol is able to accumulate in the cell membrane of *Salmonella* spp and other bacteria strains, where it can bind to hydrogen by altering the cell membrane potential and inducing a conformational and metabolic modification (decrease of ATP production) up to the time of cell death [20]. This antimicrobial activity of the *O. vulgare* EO on bacterial and fungal membranes is common to many EOs caracterised by the same amphypathic chemical compounds. Despite their strong antimicrobial action, the use of EOs in farms is limited by their poor water solubility. This characteristic makes it necessary to convey them with suitable surfactants or through biotechnological processes. The Italian product GR-OLI is a water-soluble mixture of EOs emulsified in an inert carrier additive, which is regularly authorized as additive for use in animal feed. This mixture has been compared with the activity of the *O. vulgare* OE that recently received a positive opinion from the EFSA for use in animal production. The chemical analysis of both products shows that the *O. vulgare* OE and GR-OLI have respectively three (carvacrol, p-cymene, and γ-terpinene) and eight (limonene, carvacrol, 1-8 cineol, p-cimene, linalool, terpinen-4-ol, and thymol) chemicals with a concentration >5%. Furthermore, if compared to the *O. vulgare* EO, the GR-OLI has a lower concentration of carvacrol and a higher concentration of the other terpenic molecules with known antimicrobial action. If, on the one hand, the antimicrobial action of carvacrol is well known [29,30], on the other hand, this phenolic compound is acknowledged to be potentially toxic, depending on the concentration of use [31]. For this reason, a preliminary in vitro comparison between the antimicrobial properties of *O. vulgare* EO and this commercial aromatic mixture was needed. Data show that the MIC90 of the *O. vulgare* EO is slightly lower than that of GR-OLI against the different *Salmonella* strains tested, and that the sub-MIC of *O. vulgare* EO inhibits over time the *S.* Typhimurium growth more effectively than GR-OLI. However, while the *O. vulgare* EO is only capable of disaggregating a formed biofilm, GR-OLI is simultaneously capable of inhibiting the formation of the biofilm and disaggregating the formed one at minimal concentrations potentially compatible with animal palatability. The ability to prevent the early stages of bacterial adhesion to intestinal cells is critical for the establishment of chronic colonization in animals, which are the reservoir for acute events. In this regard, data obtained from the cell adhesion assay confirmed that GR-OLI, at very low concentrations, is actually able to inhibit bacterial adhesion to the intestinal cell line Caco-2. Inhibition occurs in different ways depending on the serotype. Specifically, the monophasic *S.* Typhimurium and *S.* Infantis strains showing the greatest resistance to antibiotics were sensitive only to the higher concentration tested, while the other strains tested were sensitive to both concentrations. These activities could be useful also with animals carrying *Salmonella* spp. asymptomatically. In these animals, it is important to inhibit both the adhesion and the formation of the biofilm to prevent contamination of the carcasses at the time of slaughtering. Furthermore, data obtained from the checkerboard test indicate that GR-OLI has synergistic action with ciprofloxacin at concentrations much lower than MIC. This data identifies a possible new resource in the fight against antibiotic resistances, as it indicates the possibility of reactivating the sensitivity to ciprofloxacin with low doses of natural compounds mixed with commercial antibiotics. Moreover, given the heterogeneity of the phytocomplex of each EO, the use of concentrations lower than MIC is not currently correlated with the development of resistance [32]. This makes the use of sub-MIC of the EOs mixtures safer against the development of potential resistances.

## 4. Materials and Methods

### 4.1. Natural Substances, Antibiotics, and Reagents

*O. vulgare* L. EO and GR-OLI (by APA-CT, Forlì, Italy), a confidential solution (under patent processing) containing the 25% *v/v* of nine EOs (*Eucalyptus globulus*, *Satureja hortensis*, *Citrus aurantium* var. *dulcis*, *Thymus vulgaris*, *Melaleuca alternifolia*, *Citrus limon*, *Lavandula hybrida*, *Melaleuca cajeputi*, *Thymus capitatus*) dispersed in a surfactant (Glyceryl polyethyleneglycol ricinoleate cod. E484), admitted in animal feed, were tested against *Salmonella* spp. No preservatives or other substances were added to the mixture.

Amoxicillin/clavulanic acid, cefotaxime, and ciprofloxacin (Sigma Aldrich, St. Louis, MO, USA) were used to test their interaction with GR-OLI. C8-C40 n-alkanes mixture, p-cymene, limonene, 1,8-cineol, thymol, carvacrol, and n-hexane were purchased from Sigma-Aldrich (Milan, Italy) and used as standards. All reference standards used for GC analysis, chromatographic-grade organic solvents, and reagents were purchased from Sigma-Aldrich (Milan, Italy).

### 4.2. Bacterial Strains and Growth Media

To study the effectiveness of the natural products, 29 isolates of *Salmonella enterica* subsp. *enterica* (specifically, 17 *S. enterica* subsp. *enterica* serovar Typhimurium, of which 4 monophasic, and 12 *S.* Infantis). *Salmonella* spp. strains were isolated, during 2017, from swine and poultry intensive farms with no epidemiological correlation and provided by Istituto Zooprofilattico of Forlì (Italy). *Salmonella* spp. strains were isolated from environmental samples (faeces, boot swabs) as part of monitoring plans for the reduction of the most important public health-related *Salmonella* serovars (*S.* Typhimurium, including monophasic variants, *S.* Enteritidis, *S.* Infantis, *S.* Virchow, and *S.* Hadar) in poultry and swine farms. The detection of *Salmonella* spp. was carried out using a culture method according to Amendment 1: Annex D of EN/ISO 6579:2002 [29]. Based on this method, colonies of presumptive *Salmonella* were subcultured and their identiy was confirmed by means of biochemical tests. The pure colonies showing typical biochemical reactions for Salmonella were also tested for the presence of *Salmonella* somatic antigens (O-antigens) and flagellar antigens (H-antigens) by slide-agglutination using polyvalent antisera (BD Difco™—Becton, Dickinson and Company, Franklin Lakes, NJ, USA). Serotyping of *Salmonella* spp. strains was carried out using a slide-agglutination test following the White-Kauffmann-Le Minor scheme according to the part 3 of ISO/TR 6579-3:2014 [30]. For this purpose, a colony from a pure culture of each *Salmonella* spp. strain was cultured on nutrient agar and incubated at 37 °C ± 1 °C overnight. After the incubation, each strain was investigated for auto-agglutination by the slide-agglutination test using a 3.5% solution of sodium chloride. Once auto-agglutination was excluded, each strain was submitted to the agglutination test for serotyping the most important public health-related *Salmonella* serovars: *S.* Typhimurium (including monophasic variants), *S*. Enteritidis, *S.* Infantis, *S.* Virchow, and *S.* Hadar. For this purpose, the following somatic antisera (O-antisera) were used: O:4, O:5, O:6, O:7, O:8, O:9, and O:46 (BD Difco™—Becton, Dickinson and Company, Franklin Lakes, NJ, USA); after agglutination with the O-antisera, the agglutination with flagellar antisera (H-antisera) was performed using the following flagellar H-antisera: H:i, H:2, H:g, H:m, H:q, H:s, H:t, H:r, H:5, H:z_10_, and H:x (BD Difco™—Becton, Dickinson and Company, Franklin Lakes, NJ, USA). For biphasic H-antigens strains (e.g., *S.* Typhimurium), if one H-phase was negative, a phase inversion was carried out using the Sven Gard method according to the part 3 of ISO/TR 6579-3:2014. Based on their antigenic formula, the *Salmonella* spp. strains were identified according to the White-Kauffmann-Le Minor scheme [31]. The antigenic formula of the *Salmonella* spp. strains used in this study is summarized in Table 5. Muller Hinton medium (MH, Sigma Aldrich, St. Louis, MO, USA) was used to grow the strains at 37 °C ± 1 °C for 24 h.

### 4.3. GC-MS Analysis

Analyses were performed on a 7890A gas chromatograph coupled with a 5975C network mass spectrometer (GC-MS) (Agilent Technologies, Waldbronn, Germany). Compounds were separated on an Agilent Technologies HP-5 MS cross-linked poly–5% diphenyl–95% dimethyl polysiloxane (30 m × 0.25 mm i.d., 0.25 μm film thickness) capillary column. The column temperature was initially set at 45 °C, then increased at a rate of 2 °C/min up to 100 °C, then raised to 250 °C at a rate of 5 °C/min, and finally held for 5 min. The injection volume was 0.1 μL, with a split ratio 1:20. Helium was used as the carrier gas, at a flow rate of 0.7 mL/min. The injector, transfer line, and ion-source temperature was 250, 280, and 230 °C, respectively. MS detection was performed with electron ionization (EI) at 70 eV, operating in the full-scan acquisition mode in the m/z range 40–400. The EOs were diluted 1:20 (*v/v*) with n-hexane before GC-MS analysis.

### 4.4. GC-FID Analysis

Analyses were carried out on a gas chromatograph coupled with a flame ionization detector (FID) Agilent Technologies 7820A. Compounds were separated on an Agilent Technologies HP-5 cross-linked poly–5% diphenyl–95% dimethyl polysiloxane (30 m × 0.32 mm i.d., 0.25 mm film thickness) capillary column. The temperature programme was the same as described in Section 4.3. The injection volume was 0.1 μL in the split mode 1:20. Helium was used as the carrier gas at a flow rate of 1.0 mL/min. The injector and detector temperature were set at 250 and 300 °C, respectively. The EOs and the reference standards were diluted 1:20 (*v/v*) with n-hexane before GC-FID analysis. The analyses were performed in triplicate.

### 4.5. Qualitative and Semi-Quantitative Analysis

Compounds were identified by comparing the retention times of the chromatographic peaks with those of authentic reference standards run under the same conditions, and by comparing the linear retention indices (LRIs) relative to C8-C40 n-alkanes obtained on the HP-5 column under the above-mentioned conditions with the literature [32]. Peak enrichment by co-injection with authentic reference compounds was also carried out. Comparison of the MS-fragmentation pattern of the target analytes with those of pure components was performed, by using the National Institute of Standards and Technology (NIST version 2.0d, 2005) mass-spectral database. Semi-quantification was calculated as the relative percentage amount of each analyte; in particular, the values were expressed as the percentage peak area relative to the total composition of each EO obtained by GC-FID analysis.

### 4.6. Antimicrobial Susceptibility Testing against Antibiotics

To investigate the antimicrobial susceptibility to amicacin/clavulonic acid (AMC), piperacillin/tazobactam (TZP), cefotaxime (CTX), ceftazidime (CAZ), cefepime (FEP), ertapenem (ETP), imiprenem (IPM), meropenem (MEM), amikacin (AMK), gentamicin (GEN), ciprofloxacin (CIP), and trimethoprim/sulfamethoxazole (SXT), we performed antimicrobial susceptibility testing (AST) with the VITEK^®^ 2 system according to the manufacturer’s instructions, using the software version 7.01 and the AST-N379 cards for Gram-negative bacteria. To test the antimicrobial susceptibility against ciprofloxacin, we performed AST by the Broth Micro Dilution method according to the 2006 ISO 20776-1 procedure. MIC results were categorized as susceptible (S), susceptible by increased exposure (I), and resistant (R) according to the European Committee on Antimicrobial Susceptibility Testing (EUCAST) breakpoints (version 10.0) [33].

### 4.7. Broth Microdilution Susceptibility Testing against Natural Products

The broth microdilution (BMD) susceptibility test according to the European Committee on Antimicrobial Susceptibility Testing (EUCAST) international guidelines was performed. Muller Hilton broth (Oxoid, Basingstoke, Hampshire, UK) was used to test the antimicrobial activity of GR-OLI and *O. vulgare* EO against the *Salmonella* spp. strains. The BMD test was performed on a 96-well plate by adding 100 μL of a cell suspension equal to 5 × 10^5^ CFU/mL to a final volume of 200 μL. Scalar dilutions, between 16% *v/v* (equal to 40 μL of EOs content/ mL) and 0.125% *v/v* (equal to 0.3 μL of EOs content/mL) of GR-OLI and between 4% (40 μL /mL) and 0.03% (0.3 μL /mL) of *O. vulgare* EO, were tested. A concentration surfactant (Tween 80, Sigma Aldrich, Saint Louis, MO, USA) corresponding to that contained in the GR-OLI was tested together with *O. vulgare* EO to facilitate its solubilization in the hydrophilic medium. Plates were incubated overnight at 37 °C. After this period, MIC values were determined by spectrophotometric reading at 450 nm (EL808, Biotek, Winooski, VT, USA). To evaluate the MBC, 5 μL of the content of each well were seeded on standard medium agar plates, which were incubated for 24 h at 37 °C. Surfactants were tested separately. The MIC is defined as the lowest concentration that completely inhibits the growth of a given organism compared with the growth in the substance-free control; whereas the MBC is defined as the lowest concentration determining the death of 99.9% or more of the initial inoculum. Each test was performed in triplicate, and in each experiment suitable positive controls and blank were added. Surfactants were tested separately.

### 4.8. Biofilm Assay

All isolates were grown overnight in MH broth (Sigma Aldrich, Saint Louis, MO). To allow the formation of biofilm, cells were diluted in Luria Bertani broth (LB, Sigma Aldrich, Saint Louis, MO, USA) to a turbidity of 0.5 McFarland, corresponding to 5 × 10^8^. To study the activity of both GR-OLI and *O. vulgare* EO on the biofilm formation, both natural compounds were added in triplicate at the maximum concentration of 0.5% and 0.125% and at minimum concentration of 0.125% and 0.03%, respectively. The suspension was inoculated in polystyrene 96-well plates (Thermo Fisher Scientific, Waltham, MA, USA) and incubated at 37 °C for 48 h. No treated cells were added as a positive control in triplicate. Wells were then washed three times with PBS and the resultant biofilms were stained with crystal violet (CV) staining (Sigma-Aldrich, Saint Louis, MO, USA) for 30 min. The stained biofilms were washed in PBS and 100 μL of ethanol were added to each well for one minute to completely dissolve the CV. Then, the ethanol was transferred into a new 96-well plate to determine the absorbance at 560 nm. To test for disaggregation of biofilm, bacterial cells were prepared as described before without adding substances. After 48 h of incubation, biofilm was washed three times with PBS and cells fixated in acetone for 10 min. GR-OLI and *O. vulgare* were diluted in PBS at the maximum and minimum concentration aforementioned and added to the biofilm for another 24 h at 37 °C. Biofilm was then quantified as already described. Both tests were conducted in triplicate and repeated twice.

### 4.9. Cell Adhesion Assay

Two *Salmonella* spp. strains sensitive to almost all antibiotics (S33 and S42 resistant to only ciprofloxacin) and six multi-resistant strains (S24 and S32 *S.* Typhimurium, S19 and S29 monophasic *S.* Typhimurium and S40 and S41 S. Infantis) were randomly selected and used to study their adhesive capacity on human Caucasian colon adenocarcinoma cells (Caco-2) in the presence or absence of two concentrations of GR-OLI or *O. vulgare*.

The CACO-2 cell line was cultured in Dulbecco’s modified Eagle medium (DMEM) (Gibco, Grand Island, NY, USA) supplemented with 10% heat-inactivated fetal calf serum (Integro B.V., Zaandam, The Netherlands), 1% nonessential amino acids (Gibco, Grand Island, NY, USA), and 1 mM glutamine (Gibco, Grand Island, NY, USA) and incubated at 37 °C with 5% CO_2_. Differentiated CACO-2 cells were prepared by seeding cells 5 to 10 time in 250-mL flasks (Costar, Oneonta, NY, USA) at 1.6 × 10^7^ cells/mL in DMEM, with all supplements and then transferred to 24-well tissue culture plates at 1.6 × 10^5^ cells/mL. The culture medium was replaced every three days. Overnight grown cultures of *Salmonella* isolates were diluted (1:100) in the presence of 0.125% *v/v* and 0.5% *v/v* of GR-OIL (APA-CT, Forlì, Italy) and grown at 37 °C to an OD660 of 0.8. For each strain, an inoculum was added without adding the formulate as the control. Bacteria were harvested by centrifugation and suspended in Dulbecco′s Modified Eagle′s Medium to a final concentration of 1 × 10^7^ CFU/mL. Then, a 1-mL bacterial suspension of each strain was added to the wells (1:100 MOI). Plates were incubated for 4 h at 37 °C. After incubation, monolayers were rinsed three times with PBS (phosphate buffer solution) and cells were gently scraped with a cell scraper (Falcon, Reynosa, Tamaulipas, Mexico) and harvested with PBS and washed by centrifugation twice. The adherent bacteria were quantified by plating serial dilutions on LB agar plates and counting CFU. The inoculum was plated to determine viable counts. The assay was performed in triplicate and repeated twice.

### 4.10. Growth Curves

As described in the broth microdilution susceptibility testing method, a suspension of 5 × 10^5^ cfu/mL of the same strains used for the cell adhesion assay was seeded in a 96-well plate together with *O. vulgare* EO or GR-OLI at the MIC and sub-MIC concentrations or only with culture medium (growth control). Strains were incubated at 37 °C and monitored overnight by detecting OD450 every 30 min for 20 h. A statistical comparison between the OD450 detected at 10, 15, and 20 h of treated and untreated samples was made to quantify the extent of growth inhibition for each treatment.

### 4.11. Checkerboard Titration Method

Four strains of *S.* Infantis (S26, S35, S36, S37), all multi-resistant to amoxicillin/clavulanic acid, cefotaxime, cefepime, and ciprofloxacin, were tested using the checkerboard titration method. Then, 96-well microplates were used, each one containing MH broth with concentrations ranging from 12.5% *v/v* (equal to 31 μL of EOs content /mL) to 0.19% *v/v* (equal to 0.5 μL of EOs content /mL) for GR-OLI and from 128 to 0.125 μg/mL for amoxicillin/clavulanic acid or from 16 to 0.03 μg/mL for cefotaxime or from 4 to 0.005 μg/mL for ciprofloxacin and a combination of GR-OLI and one of the aforementioned antibiotics in a checkerboard style. The final inoculum was 5 × 10^5^ cfu/well. The microplates were incubated for 24 h at 37 °C. After the incubation period, the MBCs were evaluated by sowing 5 μL of the contents of each well on nutrient agar and incubating it at 37 °C for 24 h. The FIC value could not be evaluated as due to the turbidity of the contents of the wells, it was not possible to define the MIC values, while the FBC index were calculated in compliance with international guidelines (EUCAST, 2000). Synergism was defined as FBC index <0.5; additivity FBC index between 0.5 and 1; indifference FBC index between 1 and 2; and antagonism FBC index > 2 [33] (EUCAST, 2000). Each experiment was performed in triplicate, independently.

### 4.12. Statistical Analysis

Relative data of biofilm inhibition and disaggregation (OD values) and of cell adhesion assays (CFU values) were plotted as means ± standard errors (SE). Means whose SE bars did not overlap were considered significantly different. Relative data of growth curves at 10, 15, and 20 h after treatment with *O. vulgare* EO and GR-OLI, which were shown to satisfy the conditions for ANOVA, and were subjected to one-way ANOVA within each *Salmonella* strain and time after treatment. The lowest significant difference (LSD) test at *p* < 0.05 was used to separate levels in strain/time combinations significant at the ANOVA.

## 5. Conclusions

In conclusion, although the current European legislation admits the use of EOs as flavouring for animal foods, no previous study shows their potential ability to break down the intestinal biofilm of *Salmonella* spp. in livestock, when used at the concentrations admitted for flavouring. Furthermore, our results strongly confirm our hypothesis, since sub-MIC concentrations of both natural compounds interfere with microbial adhesion to intestinal target cells. Finally, sub-MIC concentrations of GR-OLI were able to reactivate the sensitivity of multi-resistant *Salmonella* spp. strains to ciprofloxacin, one of the most used antibiotics in veterinary practices. Our in vitro data, although needing confirmation with in vivo studies, lay the foundations for a new potential use of essential oil-based flavours in the fight against zoonosis. Indeed, although there are many studies on the antimicrobial activity of EOs, in the last 5 years, there is a paucity of articles aimed at evaluating the antimicrobial activity of single EOs on Salmonella spp. strains isolated from chicken and pig farms, and no clinical trial about the in vivo activity of EOs, or a mixture of these, on Salmonella spp. colonization has been published. In this article, for the first time, the potential preventive use of an EOs mixture used in feed as “flavourings” (GR-OLI) against *Salmonella* spp. strains directly isolated from chicken and pig farms has been shown.

## Figures and Tables

**Table 1:** Chemical composition and LRIs of natural compounds.

		%	
Components	LRI	*O. vulgare*	GR-OLI
α−thujene	927	0.82	0.13
α−pinene	934	0.98	2.48
camphene	948	0.13	0.24
sabinene	974	n.d.	0.38
β−pinene	976	0.15	2.20
octen-3-ol	980	0.46	n.d.
2-octanone	988	0.22	n.d.
β-myrcene	993	1.51	0.90
3-octanol	998	0.05	n.d.
α−phellandrene	1005	0.18	n.d.
α− terpinene	1017	1.06	1.24
p-cymene	1026	7.04	10.62
limonene	1029	0.47	15.32
1,8-cineol	1034	n.d.	11.95
cis-ocimene	1040	n.d.	1.36
trans ocimene	1049	0.05	0.22
γ−terpinene	1060	5.50	3.80
trans sabinene hydrate	1067	0.18	n.d.
cis linalool oxide	1073	n.d.	0.11
terpinolene	1089	0.15	0.63
linalool	1102	1.48	8.73
fenchol	1115	n.d.	0.21
camphor	1146	n.d.	0.45
borneol	1167	0.19	0.44
terpinen-4-ol	1179	0.53	7.12
p-cimen-8-ol	1188	n.d.	0.10
α-terpineol	1193	n.d.	2.75
linalyl acetate	1263	n.d.	5.03
thymol	1296	2.78	5.98
carvacrol	1315	66.98	12.50
neryl acetate	1369	n.d.	0.14
geranyl acetate	1387	n.d.	0.21
β−caryophyllene	1427	1.64	0.65
α-trans bergamotene	1442	n.d.	0.08
aromadendrene	1446	n.d.	0.24
β-farnesene	1462	n.d.	0.27
γ-cadinene	1512	n.d.	0.09
caryophyllene oxide	1594	0.14	n.d.
α-bisabolool	1694	n.d.	0.39

**Table 2:** Sensitivity of strains to antibiotics and natural products.

D.	Origin	Sample Source	SP.	AMC	TZP	CTX	CAZ	FEP	ETP	IPM	MEM	AMK	GEN	CIP	SXT	GR-OLI	*O. vulgare*
**3**	Swine	Feces	S.T.	S	S	S	S	S	S	S	S	S	S	R	S	4	0.25
**7**	Swine	Feces	S.T.	S	S	S	S	S	S	S	S	S	S	R	S	8	2
**12**	Swine	Feces	S.T.	S	S	S	S	S	S	S	S	S	S	R	S	8	0.5
**13**	Swine	Feces	S.T.	S	S	S	S	S	S	S	S	S	S	S	S	4	0.5
**17**	Swine	Feces	S.T.	S	S	S	S	S	S	S	S	S	R	R	S	16	2
**18**	Swine	Feces	S.T.	S	S	S	S	S	S	S	S	S	S	R	S	16	2
**21**	Swine	Feces	S.T.	S	S	S	S	S	S	S	S	S	S	R	S	8	2
**24**	Swine	Feces	S.T.	R	S	S	S	S	S	S	S	S	S	S	R	16	1
**30**	Swine	Feces	S.T.	S	S	S	S	S	S	S	S	S	S	S	S	16	0.5
**31**	Swine	Feces	S.T.	R	S	S	S	S	S	S	S	S	S	S	R	8	1
**32**	Swine	Feces	S.T.	R	S	S	S	S	S	S	S	S	R	R	S	8	1
**33**	Swine	Feces	S.T.	S	S	S	S	S	S	S	S	S	S	S	S	8	1
**34**	Swine	Feces	S.T.	R	S	S	S	S	S	S	S	S	S	S	S	2	<0.25
**19**	Swine	Feces	m.S.T.	R	I	S	S	S	S	S	S	S	S	R	S	4	1
**27**	Swine	Feces	m.S.T.	R	S	S	S	S	S	S	S	S	S	S	S	4	1
**28**	Swine	Feces	m.S.T.	R	S	S	S	S	S	S	S	S	S	R	S	16	1
**29**	Swine	Feces	m.S.T.	R	S	S	I	S	S	S	S	S	S	S	R	0.5	<0.25
**4**	Chicken	Boot swabs	S.I.	S	S	S	S	S	S	S	S	S	S	R	R	8	<0.25
**10**	Chicken	Boot swabs	S.I.	S	S	R	I	I	S	S	S	S	S	R	S	8	1
**25**	Chicken	Boot swabs	S.I.	R	S	S	S	S	S	S	S	S	S	R	R	16	1
**26**	Chicken	Boot swabs	S.I.	R	S	R	I	R	S	S	S	S	S	R	S	16	1
**35**	Chicken	Boot swabs	S.I.	R	S	R	I	R	S	S	S	S	S	R	R	4	0.5
**36**	Chicken	Boot swabs	S.I.	R	S	R	I	R	S	S	S	S	S	R	R	>16	1
**37**	Chicken	Boot swabs	S.I.	R	S	R	I	R	S	S	S	S	S	R	R	16	<0.25
**38**	Chicken	Boot swabs	S.I.	R	S	S	S	S	S	S	S	S	S	R	R	16	1
**39**	Chicken	Boot swabs	S.I.	R	S	S	S	S	S	S	S	S	S	R	R	16	1
**40**	Chicken	Boot swabs	S.I.	S	S	R	I	R	S	S	S	S	S	R	R	4	0.5
**41**	Chicken	Boot swabs	S.I.	R	S	R	I	R	S	S	S	S	S	R	R	>16	1
**42**	Chicken	Boot swabs	S.I.	S	S	S	S	S	S	S	S	S	S	R	S	16	<0.25
															**MIC90**	**16**	**2**

Note: D = Designation, SP = Species, AMC = Amicacin/Clavulonic Acid, TZP = Piperacillin/Tazobactam, CTX = Cefotaxime, CAZ = Ceftazidime, FEP = Cefepime, ETP = Ertapenem, IPM = Imiprenem, MEM = Meropenem, AMK = Amikacin, GEN = Gentamicin, CIP = Ciprofloxacin, SXT = Trimethoprim/Sulfamethoxazole, S = Sensitivity, I = Increased exposure sensitivity, R = Resistance, S.T. = *S.* Typhimurium, m.S.T. = monophasic *S.* Typhimurium, S.I. = *S.* Infantis.

**Table 3:** Relative growth of *S.* Typhimurium and *S.* Infantis at 10, 15, and 20 h after treatment with *O. vulgare* EO and GR-OLI vs. untreated control (=100).

Strain	*S.* Typhimurium				*S.* Infantis	
Product	10 h	15 h	20 h	10 h	15 h	20 h
Ctrl	100 a	100 a	100 a	100 a	100 a	100 a
*O. vulgare* EO	57 b	63 c	66 b	64 b	67 b	67 b
GR-OLI	65 b	82 b	99 a	66 b	64 b	64 b

Note. In each *Salmonella* species and time after treatment, different letters indicate significantly different means (LSD test at *p* < 0.05). Ctrl = Control.

**Table 4:** Average MBC values of amoxicillin/clavulanic acid, cefotaxime, and ciprofloxacin alone and combined with GR-OLI, and relative FBCI values.

Strain	MBC_AMC_	MBC_CTX_	MBC_CIP_	MBC_GR_	MBC_AMC/GR_	MBC_CTX/GR_	MBC_CIP/GR_	FBCI_AMC/GR_	FBCI_CTX/GR_	FBCI_CIP/GR_
26	32	>128	0.5	12.5	0.25/1.56	0.5/1.56	0.01/3.125	0.133 (s)	0.270 (s)	0.128 (s)
35	64	128	0.5	12.5	0.125/1.56	0.25/3.12	0.03/6.25	0.127 (s)	0.530 (a)	0.321 (s)
36	64	128	0.5	12.5	0.25/6.25	0.5/1.56	0.005/6.25	0.504 (a)	0.505 (a)	0.128 (s)
37	64	>128	0.5	12.5	0.125/12.5	0.25/3.12	0.03/3.125	1.002 (i)	0.280 (s)	0.251 (s)

Note. AMC = Amoxicillin/clavulanic acid, CTX = cefotaxime, CIP = ciprofloxacin, (s) = synergy, (a) = additivity, (i) = indifference.

**Table 5:** Antigenic formula of *Salmonella* serovars used in the study.

Name	O-Antigens	H-Antigens
*Salmonella* Typhimurium	1,4,[5],12	I:1,2
*Salmonella* Typhimurium monophasic variant	1,4,[5],12	i:-
*Salmonella* Infantis	6,7,14	r:1,5

Note. Numbers underlined and in square brackets are in accordance with the international nomenclature for *Salmonella* spp.
